# Methylglyoxal, the dark side of glycolysis

**DOI:** 10.3389/fnins.2015.00023

**Published:** 2015-02-09

**Authors:** Igor Allaman, Mireille Bélanger, Pierre J. Magistretti

**Affiliations:** ^1^Laboratory of Neuroenergetics and Cellular Dynamics, Brain Mind Institute, Ecole Polytechnique Fédérale de Lausanne (EPFL)Lausanne, Switzerland; ^2^Division of Biological and Environmental Sciences and Engineering, King Abdullah University of Science and TechnologyThuwal, Saudi Arabia

**Keywords:** methylglyoxal, neuron, astrocyte, triosephosphate, advanced-glycation end-products (AGEs), glutathione

## Abstract

Glucose is the main energy substrate for the brain. There is now extensive evidence indicating that the metabolic profile of neural cells with regard to glucose utilization and glycolysis rate is not homogenous, with a marked propensity for glycolytic glucose processing in astrocytes compared to neurons. Methylglyoxal, a highly reactive dicarbonyl compound, is inevitably formed as a by-product of glycolysis. Methylglyoxal is a major cell-permeant precursor of advanced glycation end-products (AGEs), which are associated with several pathologies including diabetes, aging and neurodegenerative diseases. In normal situations, cells are protected against methylglyoxal toxicity by different mechanisms and in particular the glyoxalase system, which represents the most important pathway for the detoxification of methylglyoxal. While the neurotoxic effects of methylglyoxal and AGEs are well characterized, our understanding the glyoxalase system in the brain is more scattered. Considering the high energy requirements (i.e., glucose) of the brain, one should expect that the cerebral glyoxalase system is adequately fitted to handle methylglyoxal toxicity. This review focuses on our actual knowledge on the cellular aspects of the glyoxalase system in brain cells, in particular with regard to its activity in astrocytes and neurons. A main emerging concept is that these two neural cell types have different and energetically adapted glyoxalase defense mechanisms which may serve as protective mechanism against methylglyoxal-induced cellular damage.

## Introduction

The brain has high energy requirements and glucose is the main energy substrate for the brain. About 20% of the oxygen consumed by the human body are dedicated to cerebral functions (Mink et al., 1981), yet the brain represents around only 2% of the total body mass (Molina and DiMaio, 2012). Maintenance and restoration of ion gradients dissipated by signaling processes such as postsynaptic and action potentials, as well as uptake and recycling of neurotransmitters, are the main processes contributing to the high energy needs of the awake brain (Jolivet et al., 2009; Harris et al., 2012; Hyder et al., 2013). For instance, it has been estimated that glutamate-mediated neurotransmission is responsible for most of the energy expended in the gray matter (Sibson et al., 1998; Shulman et al., 2004; Hyder et al., 2006), highlighting the close relationship between brain activity, glutamatergic neurotransmission, energy requirements and glucose utilization.

**Figure 1:**
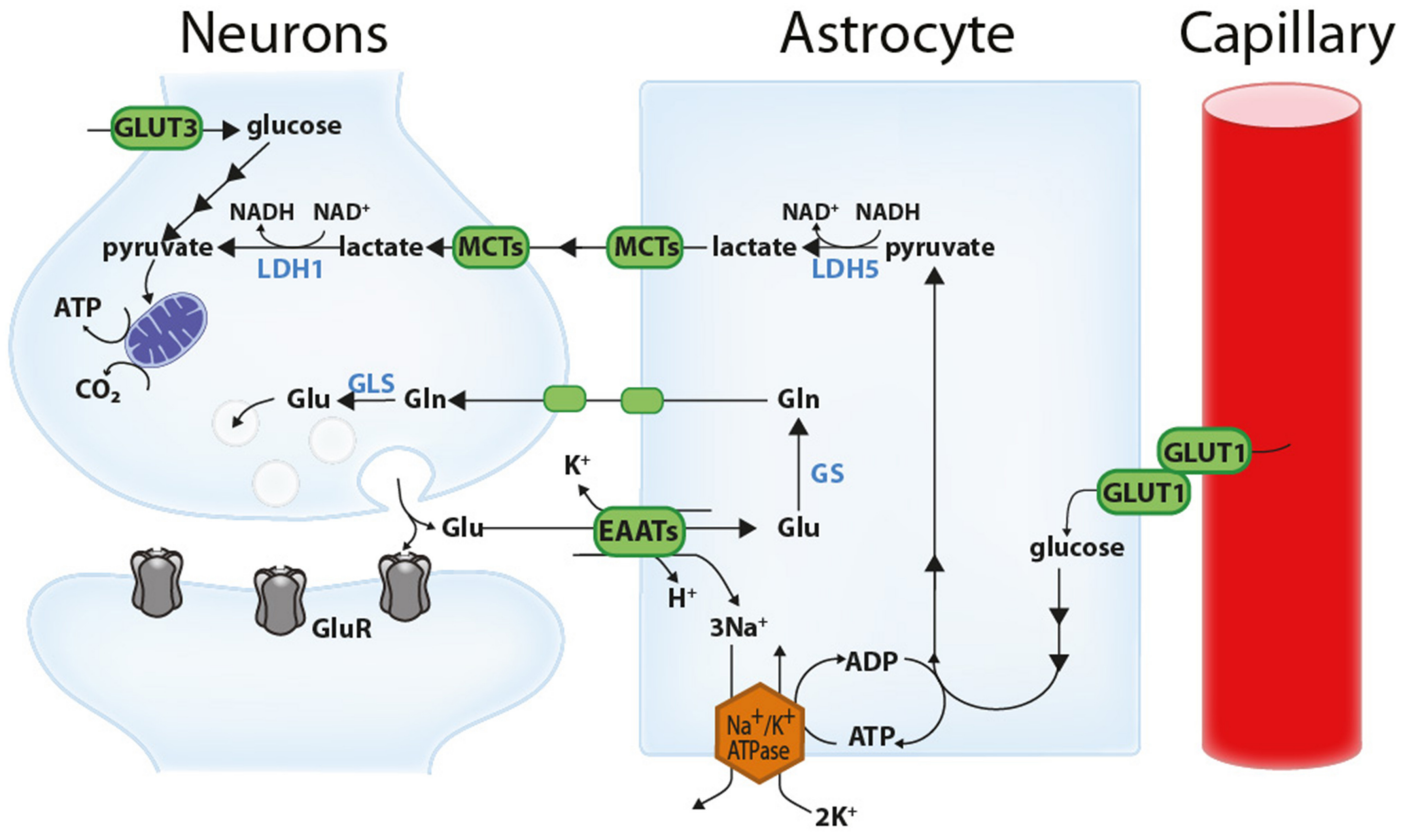
**Schematic representation of the astrocyte-neuron lactate shuttle (ANLS)**. Glutamate (Glu) released at the synapse activates glutamatergic receptors (GluR) and is associated with important energy expenditures in neuronal compartments. A large proportion of the glutamate released at the synapse is taken up by astrocytes via excitatory amino acid transporters (EAATs, more specifically GLT-1 and GLAST) together with 3 Na^+^ ions. This Na^+^ is extruded by the action of the Na^+^/K^+^ ATPase, consuming ATP. This triggers non oxidative glucose utilization in astrocytes and glucose uptake from the circulation through the glucose transporter GLUT1 expressed by both capillary endothelial cells and astrocytes. Glycolytically-derived pyruvate is converted to lactate by lactate dehydrogenase 5 (LDH5; mainly expressed in astrocytes) and shuttled to neurons through monocarboxylate transporters (mainly MCT1 and MCT4 in astrocytes and MCT2 in neurons). In neurons, this lactate can be used as an energy substrate following its conversion to pyruvate by LDH1 (mainly expressed in neurons). Neurons can also take up glucose via the neuronal glucose transporter 3 (GLUT3). Concomitantly, astrocytes participate in the recycling of synaptic glutamate via the glutamate-glutamine cycle. Following its uptake by astrocytes, glutamate is converted to glutamine (Gln) by the action of glutamine synthetase (GS) and shuttled to neurons where it is converted back to glutamate by glutaminases (GLS). Reproduced with permission from Bélanger et al. (2011a).

While neurons have high energy requirements, astrocytes are generally considered to account for around 20% of the awake brain's energy expenditure (Harris et al., 2012; Hyder et al., 2013). Intriguingly, experimental evidence demonstrates that the amount of glucose that astrocytes actually take up is disproportionally high in comparison to their energy requirements. For example, in acute cerebellar slices, the uptake of fluorescent glucose analogs is several-fold higher in Bergmann glia than in Purkinje cells (Barros et al., 2009; Jakoby et al., 2014). Nevertheless, one should mention that the stereospecificity of such fluorescent glucose analogs has been questioned (Yamamoto et al., 2011). In the resting rat brain, other studies have shown that astrocytes are responsible for approximately half of glucose uptake (Nehlig et al., 2004; Chuquet et al., 2010), and that this proportion increases even further upon functional activation (Chuquet et al., 2010). Such observations may be seen as paradoxical since neurons, not astrocytes, have the highest energy needs. A simple explanation for such an apparent paradox is the transfer of glycolysis-derived energy substrates from astrocytes to neurons. For instance, according to the astrocyte-neuron lactate shuttle (ANLS), glutamate uptake into astrocytes during synaptic activation (i.e., glutamatergic neurotransmission) stimulates glucose uptake and aerobic glycolysis in astrocytes. Lactate produced through astrocytic aerobic glycolysis is then shuttled to neurons where it is used to produce energy in the mitochondria, i.e., lactate is converted to pyruvate which is then processed in the tricarboxylic acid (TCA) cycle, hence bypassing neuronal glycolysis (Magistretti, 2009; Bélanger et al., 2011a; Pellerin and Magistretti, 2012) (Figure 1, see Figure legend for more details).

In line with this, here is now strong evidence demonstrating that cerebral glucose consumption is not homogenous for all neural cell types and that metabolic specialization takes place in terms of energy metabolism between neuron and glia (see e.g., Bélanger et al., 2011a). Consistent with their higher energy requirements, neurons sustain a high rate of oxidative metabolism compared to glial cells (Lebon et al., 2002; Itoh et al., 2003; Bouzier-Sore et al., 2006; Boumezbeur et al., 2010a). Interestingly, a large body of evidence shows that neurons can efficiently use lactate as an energy substrate (Schurr et al., 1997; Bouzier et al., 2000; Qu et al., 2000; Serres et al., 2005; Boumezbeur et al., 2010b) and even show a preference for lactate over glucose when both substrates are present (Itoh et al., 2003; Bouzier-Sore et al., 2006). By contrast, although astrocytes display lower rates of oxidative metabolism compared to neurons, they avidly take up glucose and characteristically present a high glycolytic rate (Itoh et al., 2003; Herrero-Mendez et al., 2009; Bittner et al., 2010). A large portion of the glucose entering the glycolytic pathway in astrocytes is released as lactate in the extracellular space (Pellerin and Magistretti, 1994; Itoh et al., 2003; Serres et al., 2005; Bouzier-Sore et al., 2006; Lovatt et al., 2007). Another striking difference between astrocytes and neurons is the presence of glycogen reserves almost exclusively in the glial compartment, the mobilization of which also leads to lactate release in the extracellular space (see e.g., Dringen et al., 1993; Allaman, 2009; Bélanger et al., 2011a). Even though the ANLS hypothesis is compatible with some glucose utilization by neurons, it (the ANLS) has been challenged by some studies claiming that glucose is the main, if not the exclusive, energy substrate for oxidative metabolism in neurons (see e.g., Chih and Roberts, 2003; Hertz et al., 2007; Dienel, 2012; Patel et al., 2014).

**Figure 2:**
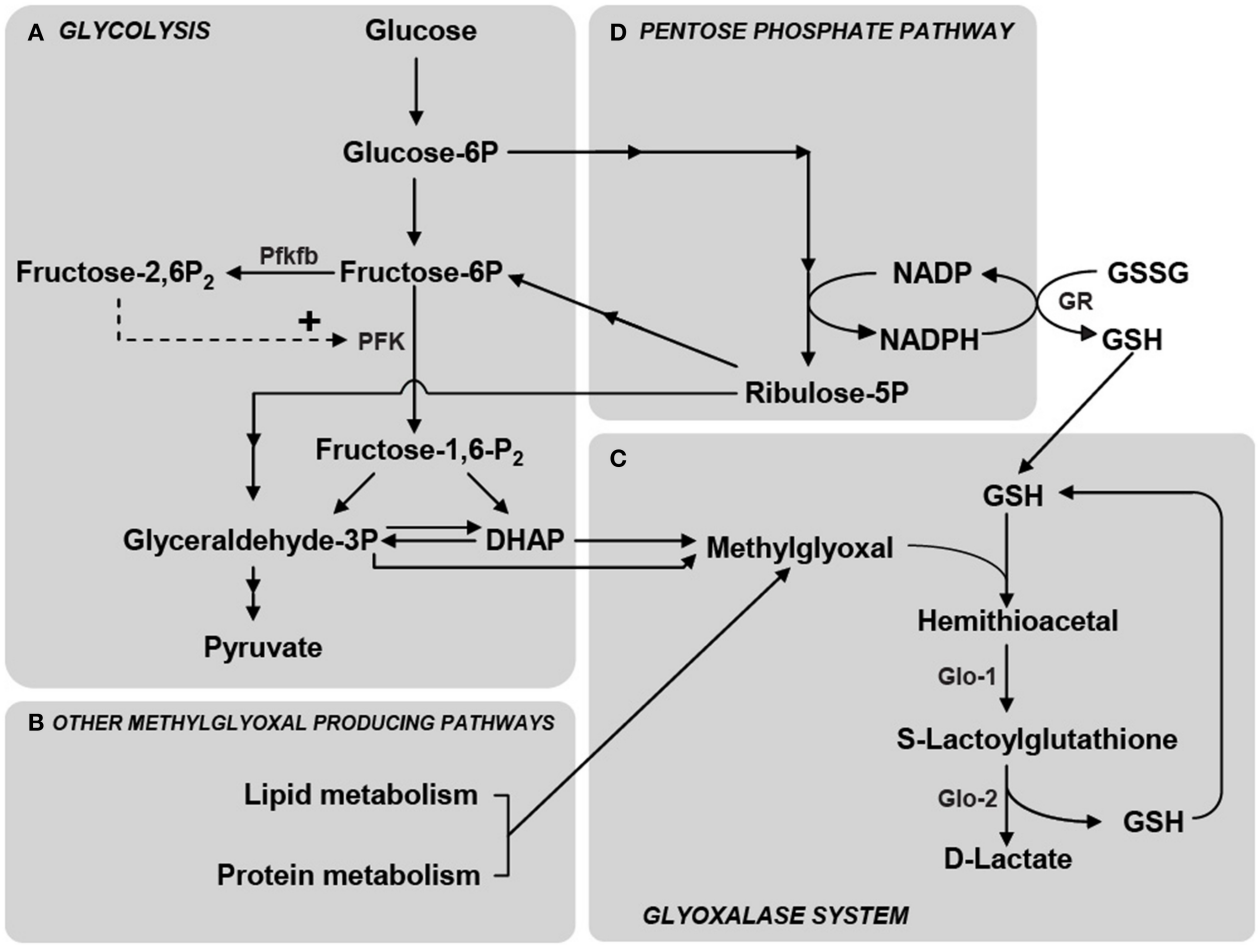
**Schematic representation of the main metabolic pathways involved in MG production and elimination. (A)** MG is formed mainly by the fragmentation of the glycolytic intermediates glyceraldehyde-3-phosphate (Glyceraldehyde-3P) and dihydroxyacetone phosphate (DHAP), but also from the metabolism of lipids and proteins **(B)**. Phosphofructokinase-1 (PFK) is the rate-limiting step of glycolysis and thus constitutes an important regulatory site, one of its most potent allosteric activators being Fructose-2,6-bisphosphate (Fructose-2,6-P_2_). Fructose-2,6-P_2_ levels are controlled by the enzyme 6-phosphofructose-2-kinase/fructose-2,6-bisphosphatase (Pfkfb) which is most abundantly expressed in astrocytes compared to neurons (Herrero-Mendez et al., 2009). **(C)** MG is detoxified principally via the glyoxalase system which consists of the enzymes Glyoxalase-1 (Glo-1) and Glyoxalase-2 (Glo-2). The first step in MG detoxification requires its spontaneous reaction with reduced glutathione (GSH) to form a hemithioacetal which is used as a substrate by Glo-1 to form S-Lactoylglutathione. Glo-2 then catalyzes the transformation of S-Lactoylglutathione into D-Lactate, recycling GSH in the process. **(D)** The pentose phosphate pathway is linked to MG detoxification via the formation of NADPH which is required for the recycling of GSH from its oxidized form (GSSG) via the action of glutathione reductase (GR). Reproduced with permission from Bélanger et al. (2011b).

In addition to this, astrocytes are known to display high metabolic plasticity in terms of glucose utilization, when compared to neurons (Pellerin and Magistretti, 1994; Almeida et al., 2001; Prapong et al., 2002; Porras et al., 2004; Gavillet et al., 2008; Allaman et al., 2010; Belanger et al., 2010). One striking example is the differential energetic response of astrocytes and neurons following the inhibition of mitochondrial respiration by nitric oxide (NO). In neurons, NO induces a massive intracellular ATP depletion and leads to apoptosis. By contrast, astrocytes respond to NO by increasing their glycolysis rate, which restrains the fall in ATP levels and prevents apoptosis (Almeida et al., 2001). A critical element accounting for the glycolytic response in astrocytes is the synthesis of fructose-2,6-bisphosphate—a potent activator of the glycolytic enzyme phosphofructokinase-1—by the enzyme Pfkfb (Almeida et al., 2004) (Figure 2). While Pfkfb is highly expressed in astrocytes, it is virtually absent in neurons due to its constant proteasomal degradation, therefore impeding glycolysis stimulation in neurons (Herrero-Mendez et al., 2009). Nevertheless, the ability of neurons to increase their glucose utilization rate in different conditions has also been described (see e.g., Dienel, 2012; Patel et al., 2014). The abovementioned observations, in conjunction with transcriptome analysis of brain cells isolated by fluorescence-activated cell sorting (FACS) revealing weaker and differential expression of glycolytic enzymes in neurons than in astrocytes (Lovatt et al., 2007; Cahoy et al., 2008; Zhang et al., 2014), sustain the view of astrocytes as a prevalent site for the glycolytic processing of glucose in the brain.

By opposition to one's common belief, glycolysis is not an innocuous metabolic pathway for cells, since it inevitably produces methylglyoxal (MG) as a by-product. The accumulation of the cell-permeant MG is highly deleterious, since this compound is one of the most potent glycating agents produced in cells. It readily reacts with proteins, lipids and nucleic acids to form advanced glycation end products (AGEs). AGEs are implicated in various pathophysiological mechanisms, including those associated with diabetic complications (cataracts, retinopathy, nephropathy, angiopathy), aging, and neurodegenerative disorders (Wautier and Guillausseau, 2001; Munch et al., 2012). In normal conditions, MG detoxification is mainly achieved by the ubiquitous enzymatic glyoxalase system that keeps MG concentration at low non-toxic cellular levels.

Since the brain has very high energy requirements in order to maintain neural cells function, brain cells are more likely to be exposed to MG compared with other cell types. Therefore, MG handling by neural cells has to be tightly regulated in order to prevent irreversible MG toxicity.

## MG and the glyoxalase system

MG, a dicarbonyl compound, is a ubiquitous product of cellular metabolism being therefore present in all cells, either under normal or pathological conditions. Enzymatic and non-enzymatic routes are known to produce MG. The rate of MG formation depends on the organism, tissue, cell metabolism and physiological conditions.

While MG can be produced as a by-product of protein and fatty acid metabolism (Thornalley, 1996; Kalapos, 1999; Vander Jagt and Hunsaker, 2003), the glycolytic pathway represents the most important endogenous source of MG via the fragmentation of the triosephosphates glyceraldehyde-3-phosphate (GAP) and dihydroxyacetone phosphate (DHAP) (Richard, 1993) (Figure 2). It is estimated that 0.1–0.4% of the glycolytic flux results in MG production (Kalapos, 2008a) and that nonenzymatic MG formation rate, from GAP and DHAP, is 0.1 mM per day in rat tissues (Richard, 1991). Cerebrospinal fluid levels of MG have been estimated to be between 10 and 20 μM (Kuhla et al., 2005), and cellular levels of free MG are typically in the low μM range (Rabbani and Thornalley, 2010). Importantly, as MG is highly reactive its half life is short in a biological environment and therefore, at the time and site of production local concentrations may be significantly higher (Kalapos, 2008b). As an example of the potency at which MG reacts with biological samples, addition of 1 μM [^14^C]MG to human plasma *ex vivo* produced complete and irreversible binding of MG to plasma protein within 24 h at 37°C (Thornalley, 2005). Consistently, up to 90–99% of cellular MG is bound to macromolecules, and assessment of total (free + bound) MG, suggested that cellular concentrations up to 300 μM can be reached (Thornalley, 1996; Chaplen et al., 1998).

High levels of MG occur when the concentrations of their precursors are elevated, such as in hyperglycemia, impaired glucose utilization and triosephosphate isomerase deficiency (Ahmed et al., 2003a).

As previously mentioned, MG is one of the most potent glycating agents present in cells making its accumulation highly deleterious. For instance, MG readily reacts with lipids, nucleic acids and with lysine and arginine residues of proteins to form AGEs such as argpyrimidine, hydroimidazolone MG-H1, MG-derived lysine dimer and N^ε^-(1-carboxyethyl)lysine (Thornalley, 2005, 2007; Rabbani and Thornalley, 2010). Besides the direct changes in protein function by MG modifications, AGE-modified proteins also exert cellular effects via their interaction with specific AGE receptors [RAGE (receptor for AGE)] (Grillo and Colombatto, 2008; Daroux et al., 2010), which triggers an inflammatory response at the cellular level, also accounting for AGE toxicity. AGEs play an important role in various pathophysiological mechanisms, including those associated with diabetic complications, aging, and neurodegenerative disorders (Wautier and Guillausseau, 2001; Ramasamy et al., 2005; Goldin et al., 2006; Munch et al., 2012).

In order to avoid the toxic effects of MG, cells possess different detoxifying mechanisms such as the glyoxalase, aldose reductase, aldehyde dehydrogenase and carbonyl reductase pathways (Thornalley, 1993; Kalapos, 1999; Vander Jagt and Hunsaker, 2003). Undoubtedly, the glyoxalase system, an ubiquitous enzymatic pathway, is the main detoxifying system for MG and other reactive dicarbonyl compounds in eukaryotic cells, thereby playing a major role the cellular defense against glycation and oxidative stress (Thornalley, 1993; Kalapos, 2008b). It detoxifies MG through two sequential enzymatic reactions catalyzed by glyoxalase-1 (Glo-1) and glyoxalase-2 (Glo-2), using glutathione as a co-factor. Glo-1 converts the hemithioacetal formed by the non-enzymatic reaction of reduced glutathione (GSH) with MG, to S-D-lactoylglutathione. This compound is then metabolized to D-Lactate (the poorly metabolizable enantiomer of L-lactate) by Glo-2, which recycles glutathione in the process (Figure 2) (Thornalley, 1993). Since S-D-lactoylglutathione is a non-toxic compound, metabolism of the dicarbonyl compound by Glo-1 represents a crucial step for MG detoxification, implying that Glo-1 activity indirectly determines MG toxicity and the rate of AGEs formation. One should also consider that GSH recycling occurs as S-D-lactoylglutathione is metabolized to D-Lactate. This implies that large increases of MG levels or low Glo-2 activity may result in S-D-lactoylglutathione accumulation, keeping GSH trapped, hence potentially leading to decreased GSH availability for other cellular processes such as defense against oxidative stress (Dringen, 2000).

## Glyoxalase system in neurons and astrocytes

Direct assessment of the intrinsic glyoxalase system capacities in both neurons and astrocytes has been done using mouse primary cortical cultures (Bélanger et al., 2011b). In this model, both Glo-1 and Glo-2 enzymes activities are significantly higher in astrocytes compared to neurons, i.e., Glo-1 and Glo-2 displayed respectively 9.8 times higher and 2.5 higher activities in astrocytes as compared to neurons.

In both cell types, Glo-1 activity rate was markedly higher compared to Glo-2 supporting the view that the rapid conversion of MG and GSH to S-D-Lactoylglutathione represents a critical step in the cellular defense against MG toxicity (Thornalley, 2008). A similar difference in the levels of Glo-1 and Glo-2 expression was also observed in C6 glioma cell lines (Hansen et al., 2012). Consistent with the differential Glo-1 activity between astrocytes and neurons, concentration-response curve analysis demonstrated that astrocytes detoxify exogenously added MG to D-lactate more efficiently than neurons (Bélanger et al., 2011b). Glo-1 enrichment in astrocytes compared to neurons was confirmed *in vivo* in the cerebral cortex of mice by immunofluorescence labeling (Bélanger et al., 2011b). Consistent with *in vitro* results, while Glo-1 immunoreactivity could be observed in both astrocytes (GFAP-positive cells) and neurons (NeuN-positive cells), the strongest levels of Glo-1 immunostaining was found in GFAP-positive cells. At the structural level, Glo-1 immunoreactivity in astrocytes was observed to be present the cell body as well as in the main astrocytic processes, i.e., along the GFAP-positive filaments.

Consistent with this observation, cell specific FACS demonstrated a 2-fold enrichment of Glo-1 activity in acutely isolated brain cortical astrocytes compared to other brain cortical cells (Bélanger et al., 2011b). Using a similar technique, Cahoy et al. (2008) demonstrated a 2.5-fold enrichment of Glo-1 mRNA expression in acutely isolated mouse astrocytes (Cahoy et al., 2008). In the human brain samples, both Glo-1 positive astrocytes and neurons were observed (Chen et al., 2004; Kuhla et al., 2006a).

Together, these results demonstrate a consistently highly efficient glyoxalase system in (cortical) astrocytes compared to neurons in both *in vitro* and *in vivo* situations. Moreover, this suggests that such a cell-specific distribution has physiological significance in the intact brain.

## Exogenous MG is highly toxic for neurons, red-ox dependency

Consistent with a less active glyoxalase system, neurons are more vulnerable than astrocytes to MG toxicity. Indeed, exogenously added MG at concentrations between 250 and 750 μM is detrimental to neuronal viability in hippocampal, cortical and primary sensory cortex cultures or in SH-SY5Y neuroblastoma cells (Di et al., 2004, 2008; de Arriba et al., 2007; Bélanger et al., 2011b; Radu et al., 2012), whereas concentrations up to 1 mM do not impact cellular integrity in astrocytic cultures or in C6 glioma cells (Bélanger et al., 2011b; Hansen et al., 2012). Direct comparison of MG toxicity in primary cultures of mouse cortical neurons and astrocytes evidenced a 6-fold higher susceptibility toward MG toxicity in neurons compared to astrocytes (Bélanger et al., 2011b). Even more strikingly, when co-cultured with astrocytes neurons are significantly protected from MG toxicity (added at concentration s of up to 2 mM) further highlighting the high capacity of astrocytes to detoxify extracellular sources of MG, protecting neurons in the process (Bélanger et al., 2011b).

Neuronal MG toxicity is associated with AGEs production and accumulation (Bélanger et al., 2011b) but is also involved in oxidative stress-mediated cell death. Indeed, intracellular glutathione and NADPH levels, as well as glutathione-dependent antioxidant processes, are strongly decreased in cultured neuronal cell preparation following MG application, leading to cellular oxidative stress and intracellular accumulation of reactive oxygen species (Di et al., 2004, 2008; de Arriba et al., 2007). As an example, co-treatment of MG with the antioxidant, and glutathione precursor, N-acetylcysteine (NAC) prevents MG-mediated neuronal death in hippocampal cultures (Di et al., 2004). This can be explained by the requirement of GSH for MG detoxification. Indeed, although GSH is recycled back during the processing of MG though the glyoxalase system at the Glo-2 step, exposure to MG is likely to cause a transient GSH depletion since part of the intracellular GSH is kept trapped in the form of S-D-lactoylglutathione during the process, especially since Glo-2 displays much slower activity rates than Glo-1 in both astrocytes and neurons (see above) (Figure 2). This transient GSH depletion resulting from glyoxalase system activity may therefore have important consequences for the cell's oxidative stress status [e.g., increasing oxidized glutathione (GSSG)/GSH and NADP/NADPH ratios], since glutathione represents one of the most important antioxidants in mammalian cells (Dringen, 2000). In line with this, GSSG was recently found to directly inactivate Glo-1 through covalent modification (Birkenmeier et al., 2010).

It has previously been reported that glutathione concentrations are higher in astrocytes than in neurons (Dringen, 2000). Such differences in glutathione metabolism, together with higher Glo-1 and -2 activities, may explain the greater resistance of astrocytes against MG toxicity compared to neurons. In agreement with this, astrocytes red-ox indexes (glutathione and NADPH levels) were not severely compromised even when cultured astrocytes were exposed to milimolar concentrations of MG (Bélanger et al., 2011b).

## Impact of manipulating Glo-1 activity in astrocytes and neurons

The observation that exogenous MG is highly damaging to neurons compared to astrocytes raises the question of the capacity of these cells to control intracellular MG production and detoxification. Experiments conducted *in vitro* on neural cells in which Glo-1 activity was decreased gave interesting clues about this question. Using RNA interference strategies Bélanger and collaborators observed that a similar decrease in Glo-1 activity (80%) does not impact astrocytic and neuronal cell viability to the same extent using primary mouse cortical cultures as a model (Bélanger et al., 2011b). For instance, Glo-1 inhibition resulted in a significant loss of neuronal viability (35%) that was associated with increased AGEs accumulation, while no deleterious effects were measured in astrocytes. Of note, due to the high Glo-1 expression levels in astrocytes, a 80% decrease in Glo-1 expression in these cells leaves Glo-1 expression at the same levels of expression than those measured in control neuronal cultures. This observation is consistent with a study conducted on SH-SY5Y cells in which chronically elevated MG concentrations induced by Glo-1 pharmacological inhibition with p-bromobenzylglutathione cyclopentyl diester (pBrBzGSCp2) also resulted in a decreased cellular viability (by 25%) (Kuhla et al., 2006b).

Considering the high susceptibility of neurons to Glo-1 silencing as described above, Bélanger et al. sought to determine whether Glo-1 upregulation could conversely protect neurons against various cellular stresses and in particular MG (Bélanger et al., 2011b). Intriguingly, they did not observed significant protection of cultured cortical neurons against exogenous MG toxicity following lentiviral-mediated Glo-1 overexpression, despite a large increase in enzymatic activity (6.8-fold increase). This result suggests that low expression level of Glo-1 is not the only factor accounting for the high neuronal vulnerability to MG toxicity. As discussed above, cultured neurons display particularly low levels of glutathione and a poor capacity to replenish the glutathione pool following exposure to MG, which may also explain neuronal susceptibility toward MG insults. Low glutathione levels may therefore represent a limiting factor for MG detoxification in neurons, in which case Glo-1 overexpression *per se* would not be sufficient to provide neuroprotective properties.

## Increased glycolysis overwhelms neuronal glyoxalase system capacity

As previously stated, the poor capacity of neurons to upregulate glycolysis (Almeida et al., 2001) is partially explained by the constitutive downregulation of Pfkfb, more specifically Pfkfb3 also known as PFK-2 isoform 3, which is responsible for the generation of fructose-2,6-bisphosphate, a key regulator of glycolysis (Herrero-Mendez et al., 2009) (Figure 2). Considering that, as described above, neurons have low MG detoxification capacity, the low protein expression levels of Pfkfb3 may be viewed as a neuronal regulatory mechanism preventing MG damages, since MG production is tightly dependent upon the glycolytic rate. In order to get insights about such hypothesis, Bélanger and collaborators manipulated glucose utilization in neuronal cultures taking advantage of the observation made by Herrero-Mendez and collaborators that overexpression of Pfkfb3 results in an increased glycolytic rate in cultured rat neurons (Herrero-Mendez et al., 2009). In primary cultures of cortical neurons, it was observed that increased lentiviral-mediated Pfkfb3 expression resulted in a limited but significant stimulation of glucose utilization (20%) (Bélanger et al., 2011b). Remarkably, this stimulation of the neuronal glycolytic rate coincided with a significant increase in intracellular MG levels and enhanced levels of argpyrimidine-modified proteins, which are MG-specific AGE product. In this context, it is worth mentioning that Herrero-Mendez and collaborators demonstrated in their initial work that an increase in neuronal glycolytic rate (i.e., induced by Pfkfb3 overexpression) was accompanied by a marked decrease in the oxidation of glucose through the pentose phosphate pathway (a metabolic route involved in the regeneration of reduced glutathione through the formation of reducing equivalent in the form of NADPH, Figure 2). Hence, less NADPH is available to regenerate the small pool of neuronal GSH, which may further compromise the efficiency of the glyoxalase system (Herrero-Mendez et al., 2009). Altogether, these results support the notion that even a modest increase in neuronal glycolytic rate can readily overwhelm the neuronal glyoxalase system, which results in the accumulation of MG and thereupon in protein damage.

## Glyoxalase system in brain disorders

Historically, most research on the glyoxalase system in diseases has focused on the importance of MG detoxification to prevent cellular damage due to the glycation of proteins and nucleic acids. These studies have implicated high concentrations of MG and/or low Glo-1 activity in the etiology of metabolic disorders, such as diabetes (Wautier and Guillausseau, 2001; Ramasamy et al., 2005; Goldin et al., 2006).

When looking more specifically at pathologies affecting the brain, modulations of the glyoxalase system have been observed in aging as well as in various neuropathological conditions, including ischemia, epilepsy, Alzheimer's disease and Parkinson's disease (Ramasamy et al., 2005; Kuhla et al., 2007; Kurz et al., 2011; Distler and Palmer, 2012). For instance, starting from the fifth decade Glo-1 expression was reported to decrease progressively in the human brain (Kuhla et al., 2006a), in correlation with increased AGEs levels in neurons and astrocytes (Luth et al., 2005). In line with this, over-expression of the Glo-1 homolog in *C. elegans* (ortholog CeGly) decreases MG-dependent toxic effects associated with the loss Glo-1 activity that occurs with age, and prolonged *C. elegans* life span (Morcos et al., 2008). Incidentally, decrease in glutathione levels during aging may also exacerbate the deleterious effects of declining Glo-1 expression (Currais and Maher, 2013). In line with this, the concentration of GSH in human lenses decreases with age (Kamei, 1993) which correlates with an increase of the MG-related AGE content (Ahmed et al., 2003b). Hence, reduced levels of glutathione in aging may not only have consequences for its function as an antioxidant, i.e., red-ox defense mechanisms, but also for its role as co-factor of the glyoxalase system (Currais and Maher, 2013).

As mentioned, impaired Glo-1 activity also contributes to the pathogenesis of neurodegenerative disorders. MG accumulation is tightly linked oxidative stress and AGE formation, which are major factors involved in many of these age-dependent pathologies. In Alzheimer's disease (AD), a marked increase in AGE accumulation is observed at later stages, which correlates with a decrease below normal levels of Glo-1 (Luth et al., 2005; Kuhla et al., 2007). In line with this, cerebrospinal fluid of patients affected by AD presents high levels of MG (Kuhla et al., 2005). A striking observation is the co-localization of AGEs with amyloid-β plaques and neurons containing hyperphosphorylated tau in AD brains, while astrocytes are also affected as they show increased AGE levels (Wong et al., 2001; Luth et al., 2005). Such observations suggest an impairment of the glyoxalase system in AD which may be involved in the vicious circle of amyloid-β deposition, AGE formation and oxidative stress associated with this disease. Nevertheless, further studies are required to delineate the specific effects of the glyoxalase system alteration on different neural cell types. Glo-1 expression was also shown to be modulated in ischemia. Indeed, following permanent middle cerebral artery occlusion, temporal and spatial changes in Glo-1 immunoreactivity in endothelial cells, neurons and astrocytes were observed (Pieroh et al., 2014).

Interestingly, higher levels of baseline MG derivatives in the serum were associated with a faster rate of cognitive decline in ederly individuals, indicating that peripheral MG metabolism imbalances may also affect brain pathopysiology (Beeri et al., 2011).

The functional defense role of the glyoxalase pathway has been exploited as a potential therapeutic target, through strategies aiming at reducing MG concentrations and/or enhancing the glyoxalase system in brain diseases. For example, More and collaborators developed a synthetic co-factor for Glo-1 (ψ-GSH) that crosses the blood brain barrier (BBB) (More et al., 2013). This molecule was shown to mitigate Alzheimer's disease indicators in a transgenic mouse model (APP/PS1). For instance, it was observed that intraperitoneal administration of ψ-GSH completely averts the development of spatial mnemonic and long-term cognitive/cued-recall impairment in these mice. Moreover, amyloid-β deposition and oxidative stress indicators were also drastically reduced in the ψ-GSH-treated APP/PS1 mouse. While a detailed characterization of the selectivity of ψ-GSH mode of action *in vivo* remains to be fully established, ψ-GSH represents a promising drug for the treatment of diseases associated with a glyoxalase dysfunction, including neurodegenerative pathologies. Increasing Glo activities in ischemia has also been shown to be an effective neuroprotective strategy. In a recent study, Glo proteins were coupled to the Tat (transactivator of transcription) peptide as a vehicle to provide Glo-1 and Glo-2 exogenously to cells of the central nervous system (Shin et al., 2014). These Tat–Glo proteins were intraperitoneally injected 30 min before performing transient forebrain ischaemia in gerbils. Increased intracerebral Glo levels were detected, indicating the ability of the agent to cross the BBB. Importantly, cell viability tests performed 7 and 14 days after ischaemia, demonstrated a neuroprotective effect of Tat-Glo proteins delivery. Interestingly, it was noted that enhancing the glyoxalase system as a whole via the simultaneous injection of Tat–Glo-1 and Tat–Glo-2 displayed the highest protection level. Such examples demonstrate the importance of the glyoxalase dysfunction in AD and ischemia, but also represent a proof of concept for the development of strategies aiming to improve the efficiency or restore the glyoxalase defense system in the context of brain disorders.

Finally, recent studies from several laboratories also suggested that modulations of MG concentration and Glo-1 activity are involved in schizophrenia, autism, anxiety, depression, sleep and pain phenotypes (Hambsch et al., 2010; Bierhaus et al., 2012; Distler and Palmer, 2012; Distler et al., 2012, 2013; Jakubcakova et al., 2013), further pointing to the role of the glyoxalase system as an important element in brain physiology and pathology.

## Physiological roles of MG and the glyoxalase system

The glutathione dependence as a co-factor for Glo-1, the non-enzymatic origin of MG from triosephosphates and the fact that the end-product is D- rather than L-lactate, isolate MG and Glo-(1 and 2) from glycolysis, leaving this system orphan in function. So is there any physiological cerebral function for this pathway, apart from its importance as a detoxifying system? There is now emerging evidence that the answer is yes. For instance, modulation of Glo-1 activity was shown to regulate anxiety-like behavior and seizure-susceptibility in mice. These effects are likely to be mediated through the regulation of MG levels, as MG was demonstrated to act as a competitive partial agonist for GABAA (γ-aminobutyric acid A) receptors (Distler et al., 2012; McMurray et al., 2014). MG was also shown to modulate the activities other proteins such as the voltage-gated sodium channel Nav1.8 or the TRPA1 ion channel (Bierhaus et al., 2012; Andersson et al., 2013). In a recent study, it was shown that D-lactate supports the viability of dopaminergic neurons *in vitro* by promoting the maintenance of mitochondrial potential (Toyoda et al., 2014). Interestingly, the latter work was linked to the discovery of DJ-1 (also known as PARK7) as a human homolog of a novel type of glyoxalase (Glo-3), that converts MG to lactic acid in the absence of glutathione (Lee et al., 2012; Toyoda et al., 2014). DJ-1 has been linked with the onset of Parkinson's disease, which is associated with mitochondrial decline in dopaminergic neurons of the substantia nigra, suggesting a possible mitochondrial regulatory role of MG metabolism in dopaminergic neurons.

## Concluding remarks

Altogether, the observations reviewed in this article support the notion that there are major differences in the glyoxalase system of astrocytes and neurons, which impact their respective capacity for cellular defense against glycation. As they are mainly based on *in vitro* observations, these findings remain to be fully established in the *in vivo* situation. Nevertheless, there is converging and convincing evidence demonstrating that astrocytes display a much more efficient glyoxalase system than neurons in cultures as well as higher Glo-1 immunoreactivity and activity in the mouse brain (Bélanger et al., 2011b). Interestingly, when compared to the high MG-producing CHO cells or various other cell types (Ranganathan et al., 1995; Shinohara et al., 1998; Wu et al., 2001; Ahmed et al., 2003a; Kumagai et al., 2009), Glo-1 activity rates in cultured astrocytic (Bélanger et al., 2011b) were shown to be several fold higher, supporting the notion of a particularly efficient glyoxalase system in astrocytes.

As previously mentioned, glutathione levels are significantly higher in astrocytes than in neurons (Dringen, 2000; Bélanger et al., 2011b). Since MG detoxification via the glyoxalase pathway requires glutathione as a co-factor, these differences in glutathione metabolism, together with a higher glyoxalase activity, endow astrocytes with a optimized glyoxalase machinery and thus a greater resistance to exogenous MG compared to neurons. Consistently, numerous studies demonstrated a high neuronal susceptibility to MG toxicity, which was associated with AGEs production and oxidative stress leading to apoptosis (Kikuchi et al., 1999; Di et al., 2004; Di Loreto et al., 2008; Chen et al., 2010; Bélanger et al., 2011b; Radu et al., 2012).

The differential Glo-1 activity observed in astrocytes and neurons may be linked to their dissimilar metabolic energetic profile. As previously mentioned, astrocytes display high metabolic plasticity in terms of glucose utilization, when compared to neurons (Pellerin and Magistretti, 1994; Almeida et al., 2001; Prapong et al., 2002; Porras et al., 2004; Gavillet et al., 2008; Allaman et al., 2010; Belanger et al., 2010). In particular, astrocytes, are able to up-regulate their glycolytic rate upon the synthesis of fructose-2,6-bisphosphate by the enzyme Pfkfb3 (Almeida et al., 2004), an enzyme that is virtually absent in neurons due to its constant proteasomal degradation (Herrero-Mendez et al., 2009). Strikingly, enhancing neuronal glycolysis via Pfkfb3 overexpression leads to oxidative stress and apoptosis (Herrero-Mendez et al., 2009). The observation that even a moderate increase in neuronal glycolysis, subsequent to Pfkfb3 overexpression, was sufficient to significantly increase MG and AGEs levels (Bélanger et al., 2011b) indicates a role of MG production in the neuronal toxicity associated with an increase in the glycolytic rate. Hence, one can conceive that neurons constitutively suppress pathways potentially leading to MG formation because of their limited capacity to face deleterious dicarbonyl stress. Similarly to Pfkfb, expression of the enzymatic machinery for glycogen synthesis is tightly suppressed in neuron, explaining the virtual absence of glycogen in this cell type. Importantly, failure to down regulate glycogen synthesis machinery results in neuronal apoptosis (Vilchez et al., 2007). Therefore, glycolysis and glycogen metabolism pathways both of which are directly linked to triosephosphates metabolism are tightly repressed in neurons and lead to apoptosis if activated.

These observations may explain why astrocyte can sustain a higher basal glycolytic rate than neurons. They may also provide insights into the reason why neurons depend on astrocytes-derived lactate to satisfy their energy needs, as proposed by the ANLS model and substantiated by several *in vitro*, *ex vivo*, and *in vivo* data (Kasischke et al., 2004; Porras et al., 2004; Rouach et al., 2008; Magistretti, 2009; Chuquet et al., 2010). Considering that glucose metabolism inevitably leads to MG formation, lactate utilization may provide a mean for neurons to limit MG toxicity by reducing their glucose utilization.

**Figure 3:**
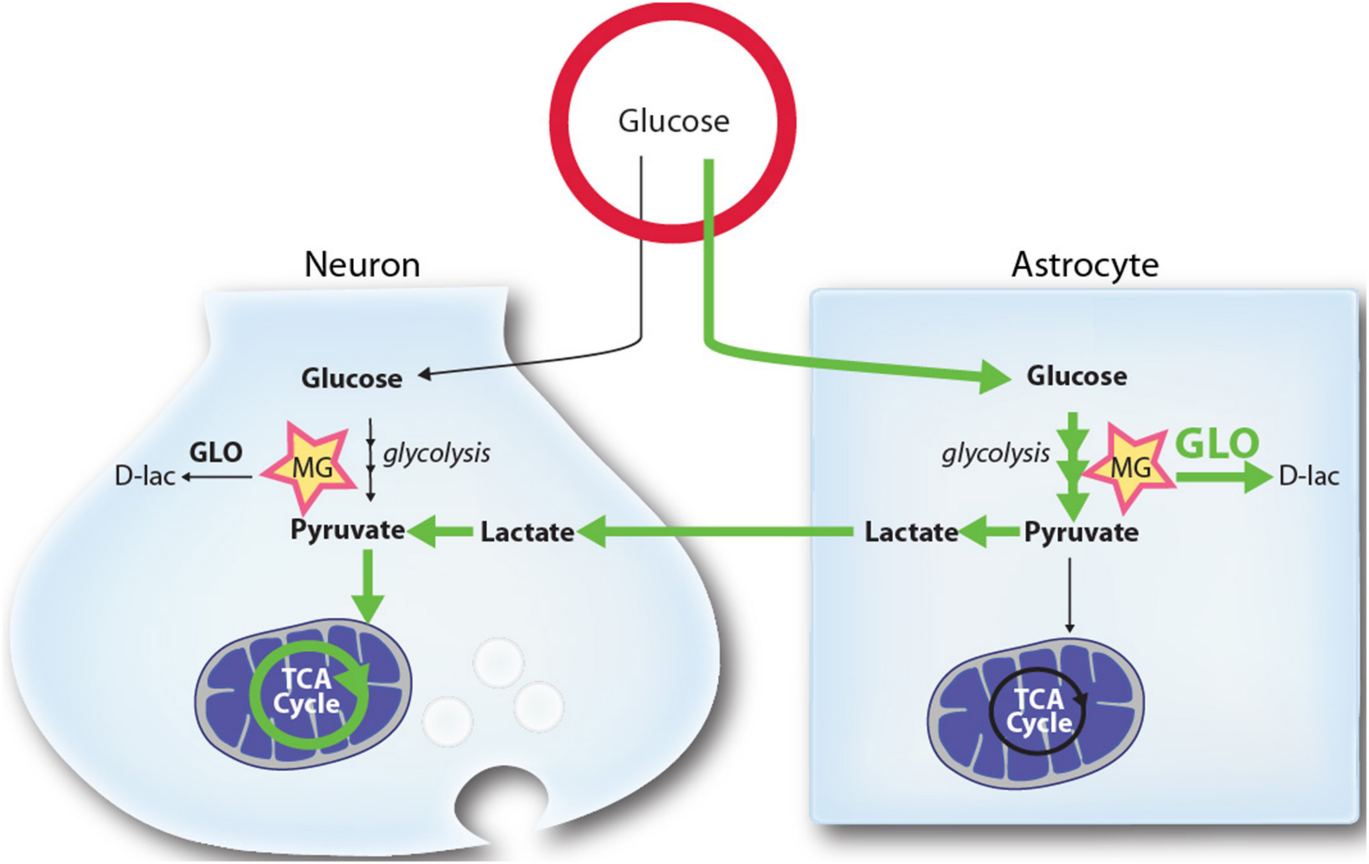
**Schematic representation of the proposed mechanism by which higher glycolytic rates in astrocytes may provide a mechanism limiting MG toxicity in neurons**. Glycolysis in both astrocytes and neurons leads to the production of the toxic MG by-product. MG is detoxified by both cell types through the glyoxalase system (GLO), producing D-lactate (D-lac). In normal and stimulated conditions (ANLS) glucose utilization and its processing through the glycolysis is tilted toward astrocytes, which release L-lactate (lactate) that can be used by neurons as a mitochondrial energy substrate. Due to the high activity of the MG-detoxifying glyoxalase system in astrocytes, these cells are well equipped to handle MG accumulation and toxicity. Because they display lower glyoxalase system activity, neurons benefit from the glycolytic processing of glucose in astrocytes since they are spared from: (1) MG accumulation and toxicity (2) alterations in their antioxidant status (through the mobilization of GSH by Glo-1), and (3) the burden of mounting an enzymatic system to process MG. See text for more details. Green arrows highlight the prevalent routes of glucose utilization in brain cells.

According to this hypothesis, neurons meet their high energetic requirements by utilizing astrocyte-derived lactate (produced from extracellular glucose or glycogen pools), which can be oxidized in the TCA cycle following its conversion to pyruvate. Such a scenario allows neurons to generate energy on demand without compromising their antioxidant status (through the mobilization of GSH by Glo-1), and leaves astrocytes with most of the MG burden (Figure 3). In line with this, the observation that astrocytes protect neurons against MG toxicity in a co-culture model (Bélanger et al., 2011b), suggests another level of astrocyte-neuron cooperativity. For instance, a highly efficient glyoxalase system in astrocytes may reinforce neuronal protection against MG potentially leaking from the periphery, the cerebrospinal fluid or surrounding cells. As a whole, such view support the concept of an “outsourced glycolysis” to astrocytes in the brain as suggested by Barros (2013).

Interestingly, evidence shows that in astrocytes, genes involved in glycogen, glycolysis and glutamate uptake are expressed through a common metabolic program (Brunet et al., 2010; Mamczur et al., 2014). Similarly, the expression of genes involved in glutathione metabolism together with Glo-1 appears to be under the control of Nuclear factor (erythroid-derived 2)-like 2 (Nrf2) that is highly expressed in astrocytes (Thimmulappa et al., 2002; Vargas and Johnson, 2009; Xue et al., 2012). This further supports the notion of a differential, complementary and coherent metabolic phenotypic differentiation between astrocytes and neurons with regards to neuroenergetics.

Consistent with its important role in controlling MG detoxification, glyoxalase system dysfunctions have been associated with various brain pathologies and in particular neurodegenerative diseases (Ramasamy et al., 2005; Kuhla et al., 2007; Kurz et al., 2011; Distler and Palmer, 2012). Astrocytes are key players in the maintenance of brain homeostasis, and several cooperative metabolic processes between astrocytes and neurons have been identified (Belanger and Magistretti, 2009; Allaman et al., 2011), which include among others energy substrate delivery (e.g., ANLS), defense against oxidative stress and excitotoxicity (Rothstein et al., 1996; Johnson et al., 2008; Vargas and Johnson, 2009; Bélanger et al., 2011a; Pellerin and Magistretti, 2012). Therefore, glyoxalase system dysfunctions may not only impact neuronal viability directly (if occurring in neurons) but also indirectly (if occurring in astrocytes) through the alterations of cooperative processes between astrocytes and neurons. As exemplified with ψ-GSH and Tat-Glo delivery in the context of AD and ischemia, strategies aiming to re-establish or boost the glyoxalase system efficiency will therefore represent potentially powerful therapeutically approaches for MG-associated brain pathologies.

It can be concluded that there is now strong evidence showing that the glyoxalase pathway differs significantly between astrocytes and neurons in a way that renders neurons more vulnerable to MG and AGE accumulation. These observations pinpoint the notion that a metabolic specialization is taking place in brain cells, i.e., astrocytes being more glycolytic than neurons, which may help protecting neurons from MG toxicity. Moreover, more than being a dead-end pathway the glyoxalase system may be important in the regulation of cerebral functions. Undoubtedly, future studies will help to shed light of the importance of this system in brain physiology and pathophysiology.

### Conflict of interest statement

The authors declare that the research was conducted in the absence of any commercial or financial relationships that could be construed as a potential conflict of interest.
